# Selection bias in clinical stroke trials depending on ability to consent

**DOI:** 10.1186/s12883-017-0989-9

**Published:** 2017-12-04

**Authors:** Benjamin Hotter, Lena Ulm, Sarah Hoffmann, Mira Katan, Joan Montaner, Alejandro Bustamante, Andreas Meisel

**Affiliations:** 1 Center for Stroke Research Berlin, Charité University Hospital Berlin, Charitéplatz 1, 10115 Berlin, Germany; 2 Department of Neurology Berlin, Charité University Hospital Berlin, Charitéplatz 1, 10115 Berlin, Germany; 3 NeuroCure Clinical Research Center Berlin, Charité University Hospital Berlin, Charitéplatz 1, 10115 Berlin, Germany; 4 Centre for Clinical Research, The University of Queensland, Herston, Queensland 4029 Australia; 5 Department of Neurology, University Hospital Zurich, Frauenklinikstrasse 26, 8091 Zurich, Switzerland; 6 Neurovascular Research Laboratory, Vall d’Hebron Institut de Recerca, Passeig Vall d’Hebron 119-129, 08035 Barcelona, Spain

**Keywords:** Stroke, Clinical trial, Informed consent, Ethics, Methods, Selection bias

## Abstract

**Background:**

Clinical trials are the hallmark of evidence-based medicine, but recruitment is often challenging, especially in stroke trials investigating patients not being able to give informed consent. In some nations, ethics committees will not approve of inclusion in a clinical study via consent of a legal representative. The ethical dilemma of including or excluding those patients has not been properly addressed, as there is little data on the effect of stroke characteristics on the ability to give informed consent.

**Methods:**

To examine differences between patients able and unable to consent at inclusion to an acute stroke trial, we conducted a post-hoc analysis of monitoring records from a multicentric interventional trial. These records listed patients who gave informed consent by themselves and those who needed a legal representative to do so. This exemplary STRAWINSKI trial aimed at improving stroke outcome by biomarker-guided antibiotic treatment of stroke associated pneumonia and included patients within 40 h after stroke onset, suffering from MCA infarctions with an NIHSS score > 9 at admission. Standard descriptive and associative statistics were calculated to compare baseline characteristics and outcome measures between patients who were able to consent and those who were not.

**Results:**

We identified the person giving consent in 228 out of 229 subjects. Patients with inability to consent were older (*p* < 0.01), suffered from more left-hemispheric (*p* < 0.01) and more severe strokes (NIHSS, *p* < 0.01), were more likely to die during hospitalisation (*p* < 0.01) or have unfavourable outcome at discharge (mRS, *p* < 0.01), to develop fever (*p* < 0.01) and tended to be more susceptible to infections (*p* = 0.06) during the acute course of the disorder.

**Conclusions:**

Demographics, stroke characteristics and outcomes significantly affect stroke patients in their ability to consent. Where selection criteria and primary outcome measures of a trial are significantly affected by ability to consent, excluding patients unable to consent might be unethical.

**Trial registration:**

URL http://www.clinicaltrials.gov. Unique identifier: NCT01264549.

**Electronic supplementary material:**

The online version of this article (10.1186/s12883-017-0989-9) contains supplementary material, which is available to authorized users.

## Background

Clinical trials often suffer from low recruitment with subsequently prolonged duration of the trials [1]. Previous work has identified special obstacles stroke research has to face. Not only is the time window of intervention limited by pathophysiological circumstances, but also, ability of patients to provide informed consent is frequently impeded due to disabilities caused by the stroke itself [2–4]. Regulatory approaches to clinical research with patients unable to give informed consent themselves differ substantially between countries [5, 6]. In the USA, regulations differ strongly between federal states, with some of them not having formal criteria of what constitutes a sufficiently authorized legal representative, leaving interpretation to investigators and ethics committees. In some countries – like Germany – the authorization to obtain informed consent by a legal representative might even be declined totally in one federal state, but granted in another. Some legislators - for instance in the USA and United Kingdom – now provide a framework for exceptions or “waivers” of consent to address this issue, frequently with a list of criteria the target population and the trial need to meet [7, 8]. Usually these include the condition studied to be acutely life-threatening, with unsatisfactory treatment options, frequently or regularly rendering the patients unable to consent. While the incapacity to consent is widely recognized as a major obstacle as well as an ethical dilemma of clinical trials in critical care, neurology and cognitive decline [9, 10], quantitative data characterising consenting and influencing parameters in these settings are very limited [11, 12]. The third international stroke trial (IST-3) reported different stroke severity and outcome based on the method of consent, but stands alone in the field of stroke research to do so [13].

This issue is of major significance, since some treatments can only be studied in patients severely affected by stroke. Patients who are only mildly affected have a higher probability to recover without any intervention [14]. The ability to consent is strongly linked to age, the severity and localisation of stroke [3, 15]. Furthermore, stroke-related complications such as post-stroke infections mainly occur in severely affected patients. Dysphagia and Central Nervous System-injury induced immune depression syndrome are the main risk factors for stroke-associated pneumonia (SAP) [16–18]. The frequency of SAP strongly correlates with the National Institute of Health Stroke Scale (NIHSS) score at admission [19].

To investigate possible selection bias introduced into clinical trials based on ability to give informed consent we analysed data from the “STRoke Adverse outcome is associated WIth NoSocomial Infections” (STRAWINSKI) trial. The trial was designed to analyse treatment-guidance for antibiotics by the use of ultrasensitive Procalcitonin (PCTus) as a marker for bacterial infections. For STRAWINSKI regulatory authorities granted permission to include patients by a legal representative (usually a next-of-kin) as well as by personal informed consent. Based on an explorative analysis we aimed at identifying differences between both groups in order to estimate the bias introduced when only including patients able to give informed consent personally.

## Methods

### The dataset from the STRAWINSKI trial

The NeuroCure Clinical Research Center Berlin initiated a multicentric, randomized, open-label treatment guidance trial on application of antibiotics with PCTus and blinded outcome assessment called “STRoke Adverse outcome is associated With NoSocomial Infections” (STRAWINSKI). Primary outcome of this trial was the proportion of patients with an mRS of 0–4 at day 90 after stroke [20]. Further information can be found in the published protocol and at clinicaltrials.gov (NCT01264549) [21]. STRAWINSKI was reviewed and approved by the appropriate ethics committees (Charité - Universitätsmedizin Berlin [reference EA1/267/10], State Medical Association of Brandenburg [reference AS 30(a)/2011], Kantonale Ethikkommission Zuerich [reference 2013–0195], Hospital Vall d’ Hebron Clinical Research Ethics Committee [reference TFS-ANT-2012-01]).

Screening and recruitment was performed at ten sites, eight of which in the federal states of Berlin and Brandenburg (Germany); one in Barcelona (Spain), and one in Zurich (Switzerland). Patients admitted to the respective emergency ward or stroke unit were screened for trial eligibility based on selection criteria listed in Table 1. If selection criteria were satisfied, and patients or their legal representative agreed to participation, patients were randomized to either standard medical care or additional daily PCTus measurements with a recommendation to treat with antibiotics in case PCTus > 0.05 ng/ml. An array of demographic parameters, clinical and paraclinical stroke characteristics and outcome information was collected. Patients were followed up for a total of 6 months with assessment of the primary endpoint (modified Rankin Scale, mRS) analysed at 3 months after stroke. For more protocol details please refer to the published trial protocol [21].

**Table 1:** Major selection criteria of the STRAWINSKI trial

Domain	Criterion
Age	≥ 18
Event to inclusion delay	≤ 40 h
Index Event	Non-lacunar MCA infarct with an NIHSS score > 9
Further criteria	- no CT evidence of intracerebral haemorrhage or lacunar infarct - informed consent strictly personal or by legal representative - no use of antibiotics within last 10 days - pre-stroke mRS of <4, life expectancy > 3 months

### Data monitoring and analysis

All collected data for the Case Report Forms were monitored. The mode of informed consent (personal by patient or by a legal representative) was assessed during monitoring in a pseudonymized fashion. We calculated descriptive statistics of patients’ demographics, their basic stroke characteristics and outcome measures. We furthermore compared the subgroups of the cohort based on their ability to consent with Fisher’s exact test, Chi-square, Mann-Whitney-U or with an independent samples t-test, and correlated ordinal variables with ability to consent using Somers’ Δ. For multivariate testing, we conducted logistic regression analysis using the stepwise backward method for variable selection to avoid omitted variable error. Statistics were calculated using SPSS (Version 23, IBM, Armonk, NY).

## Results

**Fig. 1:**
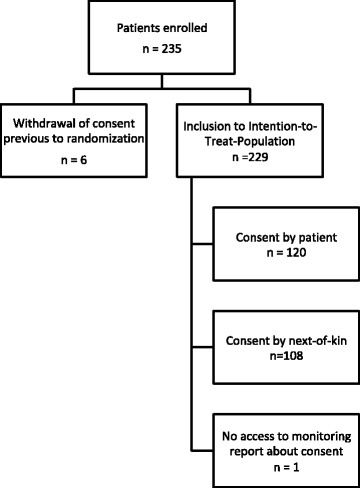
Study Flow Chart. Two patients in the Intention-To-Treat-Population violated selection criteria and were not included in the main analysis of STRAWINSKI [20]

We analysed 229 patients in the STRAWINSKI intention-to-treat-cohort (Fig. 1) [20], for all of which but one subject, information about the mode of consent (120 in person vs. 108 by legal representative) was available. Mean age of the cohort was 76.2 years ±11.3 Standard Deviation [SD], 55.5% of the participants were female. Prior to stroke most patients (75.3%) lived at home, which is reflected by a median pre-stroke mRS of 0 (Interquartile Range [IQR] 0–2). At the time of admission, patients had a median NIHSS score of 14 (IQR 12–18). A relevant proportion of 21.8% presented with a reduced state of vigilance. An infarct could be demonstrated via imaging in 96.3% during their hospitalisation. Half of the patients underwent intravenous thrombolysis (50.7%). Cardiac embolism was the most abundant cause (50.2%) for stroke in our cohort (Table 2).

**Table 2:** Basic demographics and stroke characteristics for the total cohort as well as patients able (strictly personal) and unable to consent (legal representative)

	Total cohort	Strictly personal	Legal representative	*P*-value	OR (95% CI)
n (%)	229 (100)	120 (52.6)	108 (47.4)		
Age, mean (SD)	76.2 (11.3)	73.9 (11.0)	78.9 (11.3)	0.001	t-test
Sex, % female	55.5	50.0	61.1	0.110	1.6 (0.9–2.7)
Pre-stroke mRS, median (IQR)	0 (0–2)	0 (0–1)	1 (0–3)	0.001	MWU
Pre-stroke living, n (%)				Somers‘Δ	0.304
Independent at home	171 (75.3)	102 (86.4)	68 (63.0)		
Requiring help, but at home	34 (15.0)	12 (10.2)	22 (20.4)		
Nursing or retirement home	22 (9.7)	4 (3.4)	18 (16.7)		
Admission NIHSS, median (IQR)	14 (12–18)	13 (11–15)	17 (14–21)	< 0.001	MWU
Consciousness, n (%)				Somers’Δ	0.340
Awake	179 (78.2)	107 (89.2)	71 (65.7)		
Somnolent	46 (20.1)	13 (10.8)	33 (30.6)		
Comatose	4 (1.7)	0 (0.0)	4 (3.7)		
Thrombolysis, n (%)	116 (50.7)	65 (54.2)	51 (47.2)	0.353	0.8 (0.5–1.3)
TOAST, n (%)				0.044	χ^2^
LAD	58 (26)	33 (28.2)	24 (22.9)		
CAR	112 (50.2)	51 (43.5)	61 (58.1)		
SVD	6 (2.7)	6 (5.1)	0 (0.0)		
OTH	4 (1.8)	3 (2.6)	1 (1.0)		
UNK	43 (19.3)	24 (20.5)	19 (18.1)		
Dysphagia, n (%)	164 (77)	95 (81.9)	69 (71.9)	0.100	0.6 (0.3–1.1)
Imaging proof of infarction, n (%)	181 (96.3)	91 (97.8)	89 (94.7)	0.444	0.4 (0.1–2.1)
MCA infarction left	97 (42.9)	31 (26.3)	66 (61.7)	< 0.001	4.5 (2.6–8.0)
MCA infarction right	121 (53.5)	83 (70.3)	37 (34.6)	< 0.001	0.2 (0.1–0.4)
Brainstem lesion	7 (3.1)	7 (5.9)	0 (0.0)	0.015	0.9 (0.9–1.0)
Other location	15 (6.6)	7 (5.9)	8 (7.4)	0.791	1.3 (0.4–3.6)
Randomized to PCT-guidance	112 (49.3)	57 (47.9)	55 (50.9)	0.374	0.9 (0.5–1.5)

All *p*-values given are calculated using Fisher’s exact test except where explicitly stated a Somers’ Δ correlation, Mann-Whitney-U (MWU) or an independent samples t-test; Abbreviations: *mRS* modified Rankin Scale, *NIHSS* National Institute of Health Stroke Scale score, *MCA* middle cerebral artery, *TOAST* Trial of ORG 10172 in Acute Stroke Treatment criteria, *LAD* Large Artery Disease, *CAR* cardiac embolism, *SVD* small vessel disease, *OTH* other cause, *UNK* unknown etiology

**Fig. 2:**
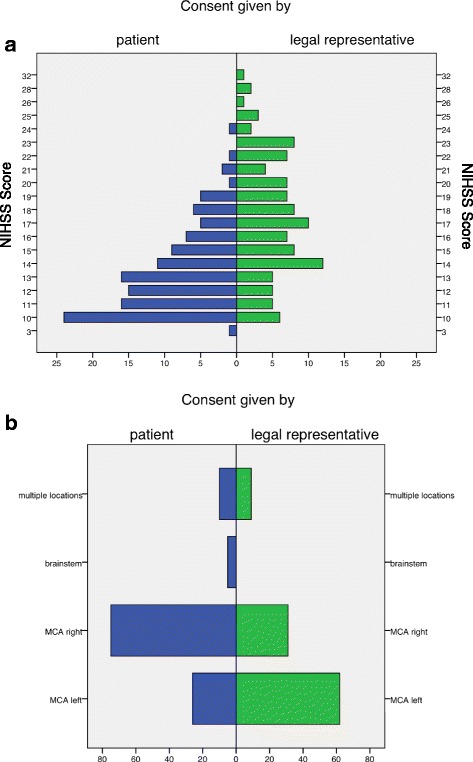
Distribution of NIHSS scores (**a**) and localization of infarction (**b**) depending on ability to consent

When looking at those measures grouped by ability to consent, the following differences appeared: Patients able to consent were younger (*p* = 0.001), had a better pre-stroke functional reserve as measured by mRS and were more likely to live at home (*p* = 0.001 and Somers’ Δ = 0.304, *p* < 0.001, respectively). They were less severely affected by stroke at admission (*p* < 0.001) measured by NIHSS scores (Fig. 2). Patients able to consent were less likely to suffer from reduced vigilance (Somers’ Δ = 0.340, *p* < 0.001). Furthermore, they had a less predominant cardioembolic etiology profile than their counterparts (*p* = 0.044). Patients with a middle cerebral artery (MCA) infarction of the left hemisphere were less likely to be able to consent (*p* < 0.001, Odds Ratio [OR] 4.5 95% Confidence Interval [CI] 2.6–8.0), whereas MCA affection of the right hemisphere was a strong positive predictor of ability to consent (*p* < 0.001, OR 0.2 95% CI 0.1–0.4). It is worth to note, that patients were evenly randomized in terms of ability to consent in the exemplary trial (*p* = 0.374, OR 0.9 95% CI 0.5–1.5).

The subjects in our cohort did persist to have a median mRS of 5 (4–5) at their time of discharge, also reflected by a low median Barthel Index (BI) of 10 (IQR 0–35). A majority of patients (72.9%) were transferred to rehabilitation centers after stroke unit care, only 7.1% were discharged home. During hospitalisation, 28.5% of patients suffered from pneumonia, 11% of urinary tract infections, 1.3% of sepsis, and 24.1% of other infections like respiratory tract infections not fulfilling criteria for pneumonia. A total of 43.6% of patients suffered from any infection. Fever was recorded in 67.3%, 33.6% showed leucocytosis and 8.5% of the patients suffered from delirium. A proportion of 10.8% required mechanical ventilation during their hospitalisation.

In terms of outcome measures, patients able to consent had a significantly better outcome at discharge measured by mRS and BI (*p* < 0.001 each). Patients unable to consent were more likely to die (*p* 0.004, OR 12.1 95% CI 1.5–96.5) and to develop fever (*p* 0.005, OR 2.3 95% CI 1.3–4.1). There was a trend to a higher rate of infections in patients unable to consent (*p* = 0.061, OR 1.7 95% CI 1.0–2.9). Further details on outcome parameters in the total cohort as well as grouped by ability to consent are given in Table 3.

**Table 3:** Outcome measures for the total cohort as well as patients able (strictly personal) and unable to consent (legal representative)

	Total cohort	Strictly personal	Legal representative	*P*-value	OR (95% CI)
Discharge mRS, median (IQR)	5 (4–5)	4 (4–5)	5 (4–5)	< 0.001	MWU
Discharge BI, median (IQR)	10 (0–35)	25 (10–40)	5 (0–20)	< 0.001	MWU
Discharge location, n (%)				Somers‘Δ	0.094
Home	16 (7.8)	8 (7.3)	8 (8.5)		
Rehabilitation hospital	164 (80.4)	92 (84.4)	71 (75.5)		
Other acute care hospital	17 (8.3)	8 (7.3)	9 (9.6)		
Nursing home	7 (3.4)	1 (0.9)	6 (6.4)		
Death in hospital, n (%)	11 (4.8)	1 (0.8)	10 (9.3)	0.004	12.1 (1.5–96.5)
mRS at 3 months, median (IQR)	4 (3–6)	4 (3–5)	5 (4–6)	< 0.001	MWU
Any SAE, n (%)	68 (29.7)	33 (27.5)	35 (32.4)	0.469	1.3 (0.7–2.2)
During hospitalisation, n (%)					
Fever	152 (67.3)	69 (59.0)	83 (76.9)	0.005	2.3 (1.3–4.1)
Delirium	19 (8.5)	8 (6.8)	11 (10.4)	0.472	1.6 (0.6–4.1)
Leukocytosis	71 (33.6)	36 (32.1)	34 (34.7)	0.770	1.1 (0.6–2.0)
Mechanical ventilation	24 (10.8)	11 (9.4)	13 (12.4)	0.521	1.4 (0.6–3.2)
Infection, n (%)	99 (43.6)	44 (37.3)	54 (50.0)	0.061	1.7 (1.0–2.9)
Pneumonia	65 (28.5)	32 (26.9)	33 (30.6)	0.560	1.2 (0.7–2.1)
Sepsis	3 (1.3)	2 (1.7)	1 (0.9)	1.0	0.5 (0.1–6.1)
Urinary tract infection	25 (11.0)	12 (10.1)	13 (12.0)	0.676	1.2 (0.5–2.8)
Other infection	55 (24.1)	26 (21.8)	28 (25.9)	0.533	1.3 (0.7–2.3)

All *p*-values given are calculated using Fisher’s exact test except where explicitly stated a Mann-Whitney-U-test (MWU) or Somers’ Δ correlation; Abbreviations: *mRS* modified Rankin Scale, *BI* Barthel Index, *SAE* serious adverse event, *PCT* procalcitonin

We then conducted a multivariate logistic regression analysis in which we included every variable that showed a *p*-value ≤ 0.1 in univariate analysis. Using the stepwise backward method we found several variables independently associated to ability to consent. These variables express severity of stroke (NIHSS score, *p* = 0.001; BI *p* = 0.059), pre-stroke functioning (living independently at home, receiving assistance at home or living in a nursing home; *p* = 0.01), location of infarction (right MCA territory, *p* < 0.001) and complications (dysphagia and fever during hospitalisation; *p* = 0.018 and *p* = 0.005, respectively) (see Table 4 for further details).

**Table 4:** Associations of demographic and stroke characteristics in patients able (strictly personal) and unable to consent (legal representative) in a multivariate logistic regression model

	*P*-value	OR (95% CI)
Pre-stroke living	0.010	2.33 (1.23–4.43)
Admission NIHSS score	0.001	1.22 (1.08–1.38)
Dysphagia	0.018	0.30 (0.11–0.82)
MCA Infarction right	<0.001	0.15 (0.06–0.37)
Discharge BI	0.059	0.98 (0.96–1.00)
Fever during hospitalisation	0.005	3.87 (1.51–9.90)

All values obtained by logistic regression with stepwise backwards exclusion. All predictors showing association of *p* ≤ 0.1 in univariate analysis were included in the analysis; Abbreviations: *NIHSS* National Institute of Health Stroke Scale, *MCA* middle cerebral artery, *BI* Barthel Index

## Discussion

In order to improve future stroke care, clinical trials will remain at the core of scientific progress. A key issue for succeeding in these is the selection of a patient population representative for “real world” stroke care. However, including patients unable to give informed consent personally is an ethically challenging issue, faced with different regulations in different countries or even between federal states [5, 6]. Some countries developed a framework by which acute treatment can be applied in emergency conditions by waiver of consent [7, 8]. Here, we explored whether exclusion of patients unable to consent would lead to a selection bias in basic demographics and classic stroke characteristics as well as in typical outcome parameters.

Our main findings are: 1) Patients able to consent were younger and had less severe strokes in term of clinical syndrome and functional deficit compared to patients unable to consent in an informed manner. 2) Location of infarction was strongly associated with the incapability to consent, most probably reflecting aphasia and unconsciousness. 3) Patients being able to consent had a higher level of pre-stroke functional independence and were more likely to live at home at the time of event. 4) Patients not being able to consent more frequently developed fever and infections and were more likely to have unfavourable outcome or even die in the course of the disorder.

These findings corroborate previous reports demonstrating stroke severity and localisation of infarction being strongly associated with ability to consent. Patients unable to consent are known to have higher admission NIHSS and mRS scores [15]. Similar to the presented study, the IST-3 investigators analysed the first 300 included patients and their consenting documents, and found stroke severity, type of clinical syndrome (motor deficit, dysphasia and/or visuospatial disorder) and infarct localization to show significant differences depending on ability to consent [13]. In a further analysis the consenting procedure was analysed in an academic stroke centre across several acute stroke studies the centre participated in. Again, greater age and greater stroke severity as measured by Scandinavian Stroke Scale were associated with inability to consent [3].

There are some limitations to this study: firstly, the results need to be interpreted with caution since we have performed this analysis in a post-hoc manner and the ability to consent was evaluated retrospectively from consenting sheets. Thus, we cannot rule out that patients gave their consent orally and next-of-kins signed as witnesses. Secondly, while we could analyse a robust sample size, larger cohorts are needed to explore further associations for different endpoints in other fields of stroke research with a higher statistical power. However, the strengths of our study are the complete data monitoring and the interventional trial setting. To our knowledge this is the second clinical trial in stroke research presenting differences within its cohort based on ability to consent, and the first to present it from its total cohort instead of an interim report.

## Conclusions

Stroke patients’ ability to consent in an informed manner to participate in a clinical study depends on several demographic factors and stroke characteristics. Location of infarction, severity of stroke and functional outcome scales differ significantly between patients able or unable to give consent. Clinical researchers investigating acute stroke in general and post-stroke immunity and infections in particular as well as ethics review boards deciding on trial protocols need to consider that patients with inability to consent are more frequently aphasic, more severely affected, older, and more likely to develop fever and infections. We recommend bearing those differences in mind when planning selection criteria for future clinical trials. If (primary) outcome measures of a trial are affected by this selection bias, it appears unethical to exclude patients unable to consent. An international political and legal framework i.e. embedded within the International Conference on Harmonisation would be immensely helpful to address this issue.

## Additional file

Additional file 1: Full dataset supporting the manuscript. (CSV 60 kb)

## Abbreviations

BI: Barthel-Index
CAR: Cardioembolism as defined by TOAST
CI: Confidence Interval
IQR: Interquartile Range
IST-3: Third international stroke trial
LAD: Large artery disease as defined by TOAST
MCA: Middle cerebral artery
mRS: modified Rankin Scale
MWU: Mann-Whitney U test
NIHSS: National Institute of Health Stroke Scale
OR: Odds Ratio
OTH: Other as defined by TOAST
PCTus: Ultrasensitive Procalcitonin
SAE: Serious Adverse Event
SAP: SAP
SD: Standard Deviation
STRAWINSKI: Stroke Adverse Outcome Is Associated with Nosocomial Infections Trial
SVD: Small vessel disease as defined by TOAST
TOAST: Trial of ORG 10172 in Acute Stroke Treatment
UNK: Unknown as defined by TOAST
